# Malignant pleural mesothelioma: treatment patterns and humanistic burden of disease in Europe

**DOI:** 10.1186/s12885-022-09750-7

**Published:** 2022-06-23

**Authors:** Adam Moore, Bryan Bennett, Gavin Taylor-Stokes, Laura McDonald, Melinda J. Daumont

**Affiliations:** 1 Adelphi Real World, Adelphi Mill, Grimshaw Lane, Bollington, Macclesfield, Cheshire SK10 5JB UK; 2 Bristol Myers Squibb, Uxbridge, UK; 3 Bristol Myers Squibb, Braine-L’Alleud, Belgium

**Keywords:** Malignant pleural mesothelioma, Disease management, Real-world, Treatment, Health-related quality of life

## Abstract

**Background:**

Malignant pleural mesothelioma (MPM) is an aggressive and rare tumour with poor prognosis. Most patients are diagnosed with advanced disease and there is a paucity of data on the humanistic burden of MPM in terms of impact on health-related quality of life (HRQoL) and activity. This study examined real-world treatment patterns and humanistic disease burden of MPM in Europe.

**Methods:**

Physicians abstracted demographic/clinical characteristics and treatment data from MPM-patient medical records; MPM patients self-completed a questionnaire including symptoms, 3-level-EQ-5D questionnaire and Visual Analogue Scale (VAS), Lung Cancer Symptom Scale for Mesothelioma (LCSS-Meso), and Work Productivity and Activity Impairment (WPAI) questionnaire.

**Results:**

Physicians (*n* = 171) abstracted data of 1390 patients; 767/1390 patients self-completed questionnaires. Patients were elderly with advanced, unresectable MPM. Treatment patterns followed guidelines with most (81%) patients receiving platinum+antifolate chemotherapy at first line (1 L). Maintenance treatment use was high (51.1%) despite no recommended maintenance therapies. Symptom burden was high and health states and HRQoL were poor at 1; declining further with progression. Overall mean (SD): LCSS-Average Symptom Burden Index score was 48.8 (19.3; *n* = 758); EQ-5D Utility Index score was 0.510 (0.349; *n* = 763); EQ-5D VAS score was 54.2 (20.3;*n* = 766); LCSS-3-Item Global Index score was 143.2 (64.5; *n* = 762); LCSS-normal activities score was 51.9 (24.6;*n* = 765); WPAI-activity impairment was 56.0% (23.2%; *n* = 737).

**Conclusion:**

The humanistic burden of MPM is high, despite treatments being prescribed as per available guidance. Treatments that delay progression and provide palliation of symptoms are most likely to improve/maintain HRQoL.

**Supplementary Information:**

The online version contains supplementary material available at 10.1186/s12885-022-09750-7.

## Background

Malignant pleural mesothelioma (MPM) is an aggressive and rare tumour, originating from mesothelial cells lining the pleura [1, 2]. MPM is the most common form of mesothelioma, accounting for over 90,000 deaths per year globally [1], with approximately 80% of cases caused by exposure to asbestos fibres [2]. The latency of MPM is approximately 40 years after asbestos exposure and prognosis is poor, with a median survival of 8–14 months from diagnosis [3], and a 5-year survival rate of 10% [4]. Long latency together with difficulty in diagnosis, due to heterogenous pathology [1, 5], diverse and non-specific signs and symptoms [3] generally means that most patients are diagnosed with advanced disease, in the metastatic state or considered unresectable, and health-related quality of life (HRQoL) is poor [6].

In Europe there has been little innovation in the management of MPM for more than a decade. The European Society for Medical Oncology (ESMO) recommends a doublet chemotherapy regimen of a platinum chemotherapy (cisplatin) + antifolate (pemetrexed) as first-line treatment (1 L) standard of care since 2004, with carboplatin and raltitrexed recommended as replacements if patients cannot tolerate cisplatin or pemetrexed, respectively [1]. There are no approvals in Europe for maintenance therapy, therapy that is administered to maintain the response achieved using 1 L systemic anti-cancer therapy (SACT), and patients are recommended to participate in clinical trials after 1 L. Surgery (maximal cytoreduction) in combination with radiation and/or chemotherapy is used in patients with early-stage disease. However, surgery alone is insufficient and radiotherapy is generally limited to adjuvant treatment following surgery and palliative care to alleviate symptoms such as pain [1].

The United States Food and Drugs Administration (FDA) recently approved the combination immunotherapy regimen of nivolumab+ipilimumab as a 1 L or second-line (2 L) treatment option following the CheckMate 743 phase 3 trial results [7]. Consequently, the National Comprehensive Cancer Network® (NCCN®) updated treatment guidelines to recommend nivolumab+ipilimumab as a preferred 1 L option with cisplatin+pemetrexed+bevacizumab also added as a preferred 1 L option [8]. Nivolumab+ipilimumab is also recommended as a preferred 2 L option, if not previously given [8]. A further immunotherapy, Pembrolizumab, is recommended at 2 L, although not in the NCCN® Guidelines. The MPM treatment landscape in Europe may soon change if recent changes in the US are reflected there.

Controlling debilitating physical symptoms such as pain, dyspnea and fatigue, and maintaining HRQoL of patients is an important consideration for the management of MPM. As disease progresses, patients experience a detrimental impact on their HRQoL [9, 10]. There is a paucity of data on the humanistic burden of MPM in terms of impact on HRQoL and activity. Given the complexity of the disease, there is a need to better understand the current management of MPM and its impact on patients in a real-world setting. We aimed to examine real-world treatment patterns of MPM and the humanistic burden of the disease.

## Methods

### Study design

This non-interventional study of MPM patients in a real-world setting included both cross-sectional and retrospective components, consisting of an electronic case report form (eCRF) completed by treating physicians and a voluntary patient self-completion questionnaire (PSC). The study was conducted in five European countries (France, Germany, Italy, Spain and the United Kingdom [UK]), representing a spectrum of different healthcare systems, between January and June 2019.

### Study population

A geographically diverse sample of physicians was recruited by local data collection agencies in each country. Specialists in medical/clinical oncology or pulmonology were included in the study if they were medically qualified for ≥5 years and < 35 years, spent at least half of their time managing patients (not limited to MPM patients), were personally responsible for drug-prescribing decisions of patients with unresectable MPM and had treated/managed ≥5 of these patients within the past 3 months.

Each physician was provided with full details of patient selection criteria and study guidelines. Physicians completed a retrospective medical chart review for the next 5–10 consectutive eligible presenting MPM patients, to mitigate against selection bias and to generate a sample representative of patients consulting in real-world clinical practice. Each eligible patient for whom a eCRF was to be completed was invited to voluntarily complete a PSC. As PSC completion was not mandatory, an opportunistic sampling approach was employed. If the patient did not complete a PSC this did not exclude their medical chart data from the medical chart review. The PSC was completed at the same visit that eligibility was determined. The PSC data provide a cross-sectional snapshot of the patients current state.

Eligible patients were ≥ 18 years old, had a physician-confirmed diagnosis of unresectable MPM, and were either receiving or had completed 1 L SACT. Those patients who had completed 1 L SACT could be receiving 2 L or later-line (2 L+) SACT or best supportive care (BSC). They were excluded if participating in a clinical trial.

### Medical chart abstraction

For each patient, physicians reviewed patients’ medical chart and abstracted patient information into eCRFs. Data collected in eCRFs included patients’ demographics, clinical characteristics (including time since diagnosis (days) and time since initiation of 1 L treatment), symptoms (at diagnosis of MPM and symptoms currently experienced at the time of data abstraction) and treatment history, including current treatment at time of data abstraction. Physician reported reasons for treatment selection were also recorded in the eCRF. The eCRF was completed online at the time of consultation, to mitigate against recall bias. There was no formal query management or resolution due to the blinded nature of the study. The eCRF included data quality control checks related to expected ranges. If the data entered were outside of an expected range the data abstractor was prompted to amend their response.

### Patient-reported outcomes (PROs)

The PSC comprised validated patient reported outcomes (PROs) including the 3-level EQ-5D questionnaire (EQ-5D-3L) and Visual Analogue Scale (EQ-5D VAS), Lung Cancer Symptom Scale-Mesothelioma (LCSS-Meso), and Work Productivity and Activity Impairment – General Health (WPAI) questionnaire. Current symptoms were also self-reported in the PSC using a pre-defined checklist of known symptoms.

The EQ-5D-3L measures participants’ general health status using 5 dimensions (mobility, self-care, usual activities, pain/discomfort, anxiety/depression). Empirically derived weights are applied to individual’s responses to EQ-5D-3L descriptive system to generate a preference-based Utility Index (UI). The UK weighting algorithm was applied to all patients for all analyses unless stated otherwise. A UI score of 1 is equal to perfect health, with lower scores indicating worse (i.e., less preferred) health states. The EQ-5D VAS records patients’ self-rated health on a 100-mm vertical VAS, from worst imaginable health (0) to best imaginable health (100) [11]. Minimally important differences, the smallest difference considered clinically meaningful, have been estimated as a difference of > 0.08 for EQ-5D UI and 7 points for EQ-5D VAS in patients with cancer [12].

The LCSS-Meso evaluates 5 major symptoms associated with lung malignancies and their impact on the average symptom burden index (ASBI; 100-mm VAS scale), and a 3-item global index (3-IGI; 300-mm VAS scale) including overall symptom severity, impact on normal activities and global HRQoL. Impact on normal activities is also presented separately (100-mm VAS scale). Higher ASBI and normal activities scores indicate greater burden; lower 3-IGI scores indicate worse HRQoL [13]. A minimally important difference (MID) of 10 points has been estimated for the ASBI [13] and 3-IGI single items [14]. When summing the 3 global items, a MID of 30 points has been used for the 3-IGI [15].

The WPAI questionnaire is a 6-item measure of the effect of a health condition on work productivity and usual activities [16]. Only the WPAI activity impairment score (expressed as an impairment percentage) is presented as most patients were no longer working due to their age.

### Data analysis

This study was descriptive in nature. Except for an exploration of differences between PSC completers and non-completers (Table 1, alpha = 0.05), inferential statistics were not used to evaluate differences between sub-groups. If an MID was available for a specific PRO measure, to aid interpretation and provide context for observed differences in PRO data between sub-groups, observed differences between sub-groups are presented with reference to this MID.

**Table 1:** Demographics and clinical characteristics of MPM patients from five EU countries (combined data)

Characteristic	Overall (***n*** = 1390)	Patients with eCRF only (***n*** = 623)	Patients with eCRF and PSC (***n*** = 767)	***p*** value (eCRF only vs eCRF and PSC)
**Country breakdown, n**				
France	365	118	168	n/a
Germany	324	137	187	
Italy	221	64	157	
Spain	241	32	209	
United Kingdom	348	202	46	
**Age, years**				
n	1390	623	767	
Age, mean (SD)	67.5 (8.7)	69.2 (8.3)	66.1 (8.8)	< 0.0001 (TT)
Age range, n^a^ (%^b^)				
0–≤64	443 (31.9)	150 (24.1)	293 (38.2)	< 0.0001 (MW)
65–74	650 (46.8)	308 (49.4)	342 (44.6)	
> 75	297 (21.4)	165 (26.5)	132 (17.2)	
**Gender, male, n (%)**	1032 (74.2)	474 (76.1)	558 (72.8)	0.1748 (FE)
**Current employment status, n (%)**				
n	1378	612	766	
Working full time	85 (6.2)	15 (2.5)	70 (9.1)	< 0.0001 (CH)
Working part time	52 (3.8)	14 (2.3)	38 (5.0)	
On long-term sick leave	201 (14.6)	83 (13.6)	118 (15.4)	
Homemaker	82 (6.0)	28 (4.6)	54 (7.0)	
Student	1 (0.1)	0	1 (0.1)	
Retired	932 (67.6)	459 (75.0)	473 (61.7)	
Unemployed	25 (1.8)	13 (2.1)	12 (1.6)	
**Current smoking status, n (%)**				
n	1368	611	757	
Current smoker	379 (27.7)	189 (30.9)	190 (25.1)	< 0.0001 (CH)
Ex-smoker	628 (45.9)	296 (48.4)	332 (43.9)	
Never smoked	361 (26.4)	126 (20.6)	235 (31.0)	
**Time since diagnosis**				
n	1310	568	742	0.0059 (TT)
Time, days, mean (SD)	298.5 (465.4)	258.1 (298.8)	329.4 (558.6)	
**ECOG PS at initial diagnosis, n (%)**^c^				
n	1374	613	761	
0	433 (32.2)	184 (30.0)	259 (34.0)	0.0228 (MW)
1	686 (49.9)	304 (49.6)	382 (50.2)	
2	225 (16.4)	113 (18.4)	112 (14.7)	
3	17 (1.2)	9 (1.5)	8 (1.1)	
4	3 (0.2)	3 (0.5)	0 (0.0)	
**Current ECOG PS, n (%)**				
n	470	223	247	
0	117 (24.9)	40 (17.9)	77 (31.2)	< 0.0001 (MW)
1	230 (48.9)	109 (48.9)	121 (49.0)	
2	108 (23.0)	61 (27.4)	47 (19.0)	
3	12 (2.6)	10 (4.5)	2 (0.8)	
4	3 (0.6)	3 (1.3)	0 (0.0)	
**Stage of MPM at diagnosis, n (%)**				
n	1380	618	762	
Stage 1	2 (0.1)	1 (0.2)	1 (0.1)	0.0974 (CH)
Stage 1a	7 (0.5)	5 (0.8)	2 (0.3)	
Stage 1b	38 (2.8)	19 (3.1)	19 (2.5)	
Stage 2	94 (6.8)	38 (6.1)	56 (7.3)	
Stage 3	303 (22.0)	142 (23.0)	161 (21.1)	
Stage 4	917 (66.4)	399 (64.6)	518 (68.0)	
Unable to stage	19 (1.4)	14 (2.3)	5 (0.7)	
**Current stage of MPM, n (%)**				
n	1328	618	710	
Stage 1	3 (0.2)	2 (0.3)	1 (0.1)	0.0007 (CH)
Stage 1a	2 (0.2)	1 (0.2)	1 (0.1)	
Stage 1b	18 (1.4)	12 (1.9)	6 (0.8)	
Stage 2	57 (4.3)	38 (6.1)	19 (2.7)	
Stage 3	235 (17.7)	110 (17.8)	125 (17.6)	
Stage 4	991 (74.6)	438 (70.9)	553 (77.9)	
Unable to stage	22 (1.7)	17 (2.8)	5 (0.7)	
**Histology of MPM, n (%)**				
n	1349	604	745	
Epithelioid	968 (71.8)	420 (69.5)	548 (73.6)	0.0775 (CH)
Biphasic	253 (18.8)	115 (19.0)	138 (18.5)	
Sarcomatoid	128 (9.5)	69 (11.4)	59 (7.9)	
**Resection status at diagnosis, n (%)**				
n	1363	615	748	
Resectable	77 (5.6)	19 (3.1)	58 (7.8)	0.0002 (FE)
Unresectable	1286 (94.4)	596 (96.9)	690 (92.2)	
**History of asbestos exposure, n (%)**				
n	1192	574	618	
Yes	897 (75.3)	476 (82.9)	421 (68.1)	< 0.0001 (FE)
No	295 (24.7)	98 (17.1)	197 (31.9)	

*Note:* EU countries include France, Germany, Italy, Spain, and the UK

*eCRF* Electronic case report form; *ECOG PS* Eastern Cooperative Oncology Group performance status; *MPM* Malignant pleural mesothelioma; *PSC* Patient self-completion questionnaire; *SD* Standard deviation

^a^ Throughout this table *n* = n minus unknown/missing

^b^Percentages may not total 100 due to rounding. n is used throughout this table as the denominator for percentages

^c^ 0 = Fully active, able to carry on all pre-disease performance without restriction.1 = Restricted in physically strenuous activity but ambulatory and able to carry out work of a light or sedentary nature, e.g., light house work, office work. 2 = Ambulatory and capable of all selfcare but unable to carry out any work activities; up and about more than 50% of waking hours. 3 = Capable of only limited selfcare; confined to bed/chair > 50% of waking hours. 4 = Completely disabled; cannot carry on any selfcare; totally confined to bed/chair

TT = Student’s t-test, MW = Mann-Whitney U, FE = Fisher’s Exact, CH = Pearson’s Chi-Squared

Continuous numerical variables were described using means and standard deviations (SD), and categorical variables were described using counts and proportions of respondents. All data analysis was undertaken in Stata v16 (2019, StataCorp. College Station, TX: StataCorp LLC).

Where a variable contained missing values on eCRFs (i.e., “don’t know” response), no imputation methods were applied to missing data. Missing data were excluded from the analysis of that endpoint, therefore the base of patients for analysis could vary from variable to variable and are reported separately for each analysis, with outcomes calculated based on patients with non-missing values for each item.

PRO data were stratified by patients’ current line of therapy (1 L, 1 L maintenance therapy [1 L-M], SACT at 2 L+, and BSC [all patients receiving BSC independent of how many previous lines of therapy they had received]). PRO data were also stratified by patients’ current treatment at time of data abstraction, including doublet chemotherapy (Group 1), triplet chemotherapy (Group 2), singlet chemotherapy (Group 3, including singlets given as 1 L-M or SACT), ‘other’ therapies (Group 4) and BSC (Group 5) (Supplementary Table 1). Data were also stratified by country and histology (epithelioid, biphasic, sarcomatoid and unknown) (Supplementary data). The demographic and clinical characteristics of patients are described for each strata in Supplementary Table 4.

### Ethical considerations

The study was approved by the Western Institutional Review Board (IRB Number: 20183141), an international review board that conducts independent ethical review of scientific research across many disciplines. Physicians and patients participated voluntarily. Patients’ decision not to complete PSCs did not disqualify their data from being recorded on eCRFs or their inclusion in the analysis. Patients providing data directly gave their informed consent prior to participating. Physician participation was financially incentivized, with reimbursement upon survey completion according to fair market research rates. Patients were not compensated for participation.

All participants’ identities were blinded from the data collection team and no patient identifiers were collected.

## Results

### Study population

In total, 171 physicians abstracted data of 1390 MPM patients. Of those, 767 patients completed PSCs (55.2% response rate). Primary specialities of physicians were oncologist (*n* = 149, 87.1%) and pulmonologist (*n* = 22, 12.9%).

The overall patient population (*n* = 1390) consisted of patients from France (*n* = 356), Germany (*n* = 324), Italy (*n* = 221), Spain (*n* = 241), and the UK (*n* = 348). A mean (SD) age of 67.5 (8.7) years was observed and approximately two-thirds were aged ≥65 years (Table 1). The majority of patients were male (74.2%), had a history of asbestos exposure (64.5%), currently smoked or were ex-smokers (72.4%), and were currently either unemployed, retired or on long-term sick leave (83.3%). Mean time since diagnosis was 298.5 days. At the time of diagnosis, patients had stage 3 or 4 (87.8%), unresectable (92.5%) disease. The majority of patients had an Eastern Cooperative Oncology Group performance status (ECOG PS) of 0, 1 or 2 at diagnosis (96.8%). At data abstraction, Mean (SD) time (days) since initiation of 1 L treatment was 201.3 (224.5) days, 88.2% of patients had stage 3 or 4 MPM, and 89.6% had an ECOG PS of 0, 1 or 2. Most patients had epithelioid MPM (*n* = 968, 69.6%); fewer patients had biphasic (*n* = 253, 18.2%), sarcomatoid (*n* = 128, 9.2%) and MPM of unknown histology (*n* = 41, 3.0%). Mean (SD) time (days) since initiation of 1 L treatment was 103.9 (89.5) days for patients currently at 1 L at data abstraction, 374.4 (229.5) days for patients at 1 L-M, 472.6 (340.8) days for patients at 2 L+ SACT and 322.9 (238.4) days for patients receiving BSC.

A comparison of the PSC completers (*n* = 767) and eCRF only patients (*n* = 623) is included in Table 1. The PSC completers group provided a large, heterogenous sample of MPM patients. Several statistically significant differences were observed between PSC completers and CRF only patients. As compared to eCRF only patients, the PSC completer group were younger (mean age of 66.1 vs 69.2 years), were more likely to be employed (14.1% vs 4.8% were full or part time employed), included fewer current smokers (25.1% vs 30.9%), had experienced a longer time since diagnosis (mean = 329.4 days vs 258.1 days), had better performance status at diagnosis (84.2% vs 79.6% with ECOG ≤1), better performance status currently (80.2% vs 66.8% with ECOG ≤1), fewer were unresectable at diagnosis (96.9% vs 92.2%) and more were currently stage 4 (77.9% vs 70.9%).

### Treatment of unresectable malignant pleural mesothelioma

#### First-line (1 L) and first line maintenance (1 L-M) treatment

At 1 L, 1127 patients (81.1%) received a platinum+antifolate doublet chemotherapy, with > 70% of patients receiving this doublet in all countries (Italy 71.9%; France 72.5%; Germany 83.0%; Spain 85.9%; UK 94.4%) and > 78% of patients across all MPM histological subtypes. Overall, 45.0% of patients received cisplatin+pemetrexed/pemetrexed disodium, and 35.5% of patients received carboplatin+pemetrexed/pemetrexed disodium. Seven patients (< 1%) received a platinum+antifolate combination that included raltitrexed.

Treatments received at 1 L by the remaining 263 patients (18.9%) varied widely, with 8.6% of patients receiving a singlet chemotherapy and 4.0% of patients receiving a triplet chemotherapy including a platinum+antifolate+other treatment. A triplet chemotherapy regimen of platinum+pemetrexed+bevacizumab was received by 44 patients (3%), with most use of this regimen observed in France (*n* = 26; 7%) and Germany (*n* = 12; 4%). The remaining 6.3% of patients received a wide range of other treatment regimens, mostly platinum+gemcitabine or vinorelbine.

**Fig. 1:**
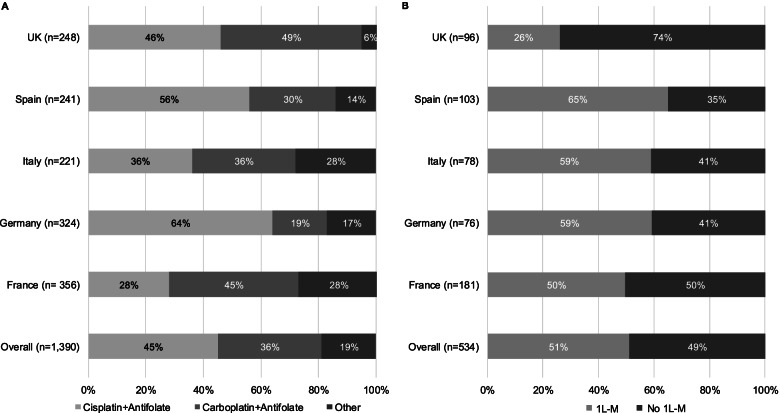
1 L combinations (**A**) and use of 1 L-M (**B**) for MPM patients, stratified by country. MPM, malignant pleural mesothelioma; 1 L, first-line therapy. Error bars represent 95% confidence intervals. ^†^Proportion of patients that had completed 1 L and went on to received 1 L-M

Of 534 patients who had completed 1 L, 273 patients (51.1%) had received/were receiving 1 L-M at data abstraction; 76.9% of these patients were receiving pemetrexed as a singlet chemotherapy. One quarter (26.0%) of UK patients that had completed 1 L received 1 L-M compared with half or more patients in each of the other countries (France 49.7%; Germany 59.2%; Italy 59.0%; Spain 65.0%; Fig. 1).

Reasons for 1 L and 1 L-M selection are included in Supplementary Table 2.

#### Second-line (2 L) and beyond

Of those patients who had started 2 L (*n* = 395), 213 patients (53.9%) received SACT at 2 L, with most patients (62.4%) receiving a singlet chemotherapy (mostly gemcitabine and/or vinorelbine). Alone or in combination treatment, gemcitabine (*n* = 87, 40.8%) was the most prescribed 2 L, followed by vinorelbine (*n* = 60, 28.2%), carboplatin (*n* = 37, 17.4%) and immunotherapies (*n* = 31, 14.6%).

Of those patients who completed 2 L (*n* = 67), 24 patients (35.8%) were receiving SACT as third-line therapy (3 L). The most prescribed 3 L was vinorelbine (45.8%), immunotherapy (25%) and gemcitabine (alone or in combination therapy, 12.5%).

In total, 230 patients had received BSC; of these, 79.1% of patients (*n* = 182) received BSC after 1 L, 18.7% of patients (*n* = 43) after 2 L and 2.2% of patients (*n* = 5) after 3 L. The most commonly prescribed BSC therapy was an opioid (62.2%), followed by pleural aspiration/drainage (32.2%), analgesics other than opioids (30.9%) and radiotherapy (21.3%).

### Current treatment at time of data abstraction of patients who completed the self-completion questionnaire (PSC)

At the time of data abstraction, of 767 patients that completed a PSC, 399 patients (52.0%) were on a platinum+antifolate doublet chemotherapy, 29 patients (3.8%) on a triplet consisting of a platinum+antifolate+other, 186 patients (24.3%) on a singlet chemotherapy, 59 patients (7.7%) on other treatments and 94 patients (12.3%) were currently receiving BSC.

### Radiotherapy and surgery

In total, 309 (22.2%) and 177 (12.7%) patients had received radiotherapy and surgery for MPM treatment, respectively; 947 patients (68.1%) had neither received radiotherapy or surgery.

### Humanistic burden of malignant pleural mesothelioma

Humanistic burden by current treatment and treatment line at time of data abstraction is presented below. Results per country and MPM histological subtype are shown in Supplementary Figs. 1 and 2.

### Symptom burden

**Fig. 2:**
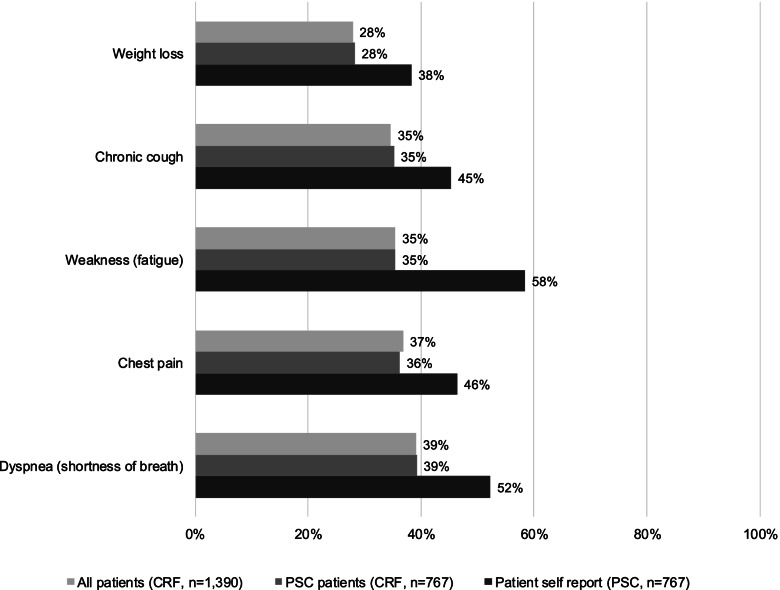
Symptoms currently experienced by MPM patients: CRF symptom data for all patients, CRF symptom data for those patients that completed the PSC, and patient self-reported symptoms recorded on the PSC. *Note:* Most frequently reported symptoms recorded for all patients. Error bars represent 95% confidence intervals. CFR, case report form; MPM, malignant pleural mesothelioma; PSC, patient self-completion questionnaire

Figure 2 demonstrates that in the overall patient population (*n* = 1390), the most common chart-abstracted symptoms experienced were dyspnea (39.1%), chest pain (36.8%), weakness (fatigue; 35.5%), chronic cough (34.7%) and weight loss (27.9%). Figure 2 also includes chart-abstracted symptoms for PSC completers, with similar proportions of patients experiencing these symptoms. It further demonstrates that more patients self-reported experiencing these common symptoms than was documented by their physicians. Tiredness/fatigue was the most common current symptom self-reported by patients.

**Fig. 3:**
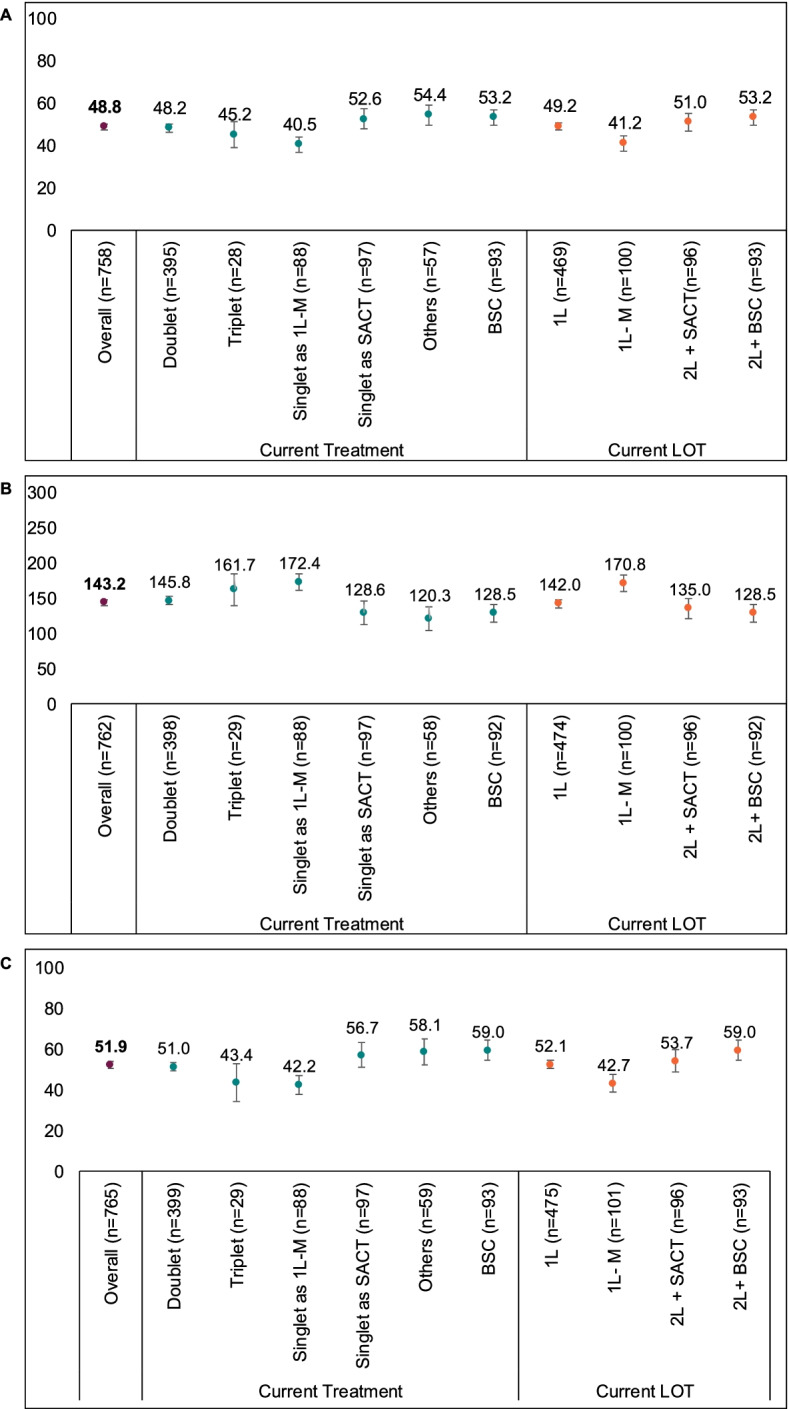
Mean LCSS ASBI (**A**), Mean LCSS-3-IGI (**B**), and mean Overall Impact on Normal Activities (**C**) of MPM patients, stratified by current treatment and by current line of therapy at time of data abstraction. *Note:* Patients from EU countries including France, Germany, Italy, Spain, and the UK. Error bars represent 95% confidence intervals. The MID for the LCSS ASBI is 10 points and the MID for the LCSS-3-IGI is 30 points. ASBI, average symptom burden index; BSC, best supportive care; doublet, doublet chemotherapy; LCSS, Lung Cancer Symptom Scale-Mesothelioma; LOT, line of therapy; MPM, malignant pleural mesothelioma; others, other therapies; SACT, systemic anti-cancer therapy; triplet, triplet chemotherapy; 1 L, first-line therapy; 1 L-M, first-line maintenance therapy; 2 L + SACT, SACT at second or later lines; 2 L+ BSC, BSC at second or later lines; 3-IGI, three-item global index

For patients with available LCSS-ASBI data (*n* = 758), the mean (SD) score was 48.8 (19.3) (Fig. 3A). Patients currently receiving singlet chemotherapy as 1 L-M had the lowest mean LCSS-ASBI score (Fig. 3A). Differences observed between LCSS-ASBI scores for this group and those receiving a singlet chemotherapy as SACT, other treatments and BSC all exceeded the MID threshold of 10. Regarding current therapy line (Fig. 3A), 1 L-M had a LCSS-ASBI score that was lower than that for BSC, with the difference exceeding the MID threshold. No clinically meaningful differences were observed between patients receiving 1 L SACT and 2 L+ SACT.

### Health-related quality of life (HRQoL)

**Fig. 4:**
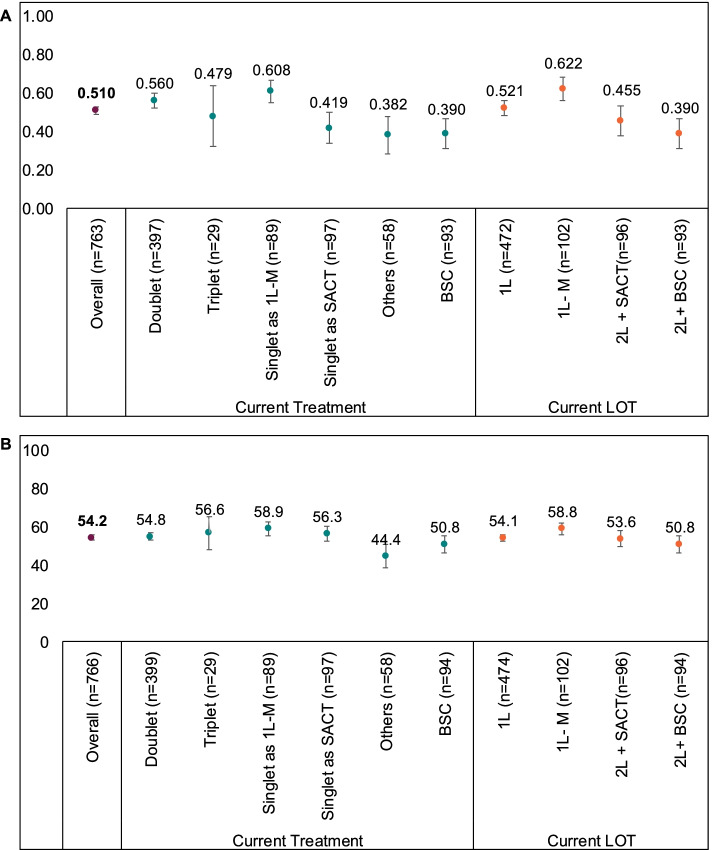
Mean EQ-5D UI (**A**) and mean EQ-5D VAS scores (**B**) of MPM patients, stratified by current treatment and by current line of therapy at time of data abstraction. *Note:* Patients from EU countries including France, Germany, Italy, Spain, and the UK. Error bars represent 95% confidence intervals. The MID for the EQ-5D UI score is 0.08 points, and the MID for the EQ-5D VAS score is 7 points. BSC, best supportive care; doublet, doublet chemotherapy; EQ-5D, European quality of life–5 dimensions; UI, utility index; LOT, line of therapy; MID, minimally important difference; MPM, malignant pleural mesothelioma; others, other therapies; SACT, systemic anti-cancer therapy; triplet, triplet chemotherapy; VAS, visual analogue scale; 1 L, first-line therapy; 1 L-M, first-line maintenance therapy; 2 L + SACT, SACT at second or later lines; 2 L+ BSC, BSC at second or later lines

For patients with available EQ-5D UI data (*n* = 763), the overall mean (SD) EQ-5D UI was 0.510 (0.349) (Fig. 4A). Differences in EQ-5D UI were observed across current therapy, with patients receiving singlet chemotherapy as 1 L-M and doublet chemotherapy having the highest EQ-5D UI (Fig. 4A). Differences observed between these groups and other groups (singlet at other lines, triplet chemotherapy, other treatments and BSC) exceeded the MID threshold.

Similar differences in EQ-5D UI were also observed in current line of therapy, with 1 L and 1 L-M patients having clinically meaningful higher EQ-5D UI than 2 L+ patients receiving SACT or BSC (Fig. 4A). Supplementary Fig. 1A includes EQ-5D UI data for each country, derived using country-specific scoring algorithms (i.e., French patients’ EQ-5D UI was derived using the French algorithm).

The EQ-5D profile showed that 83% of patients reported experiencing at least moderate pain/discomfort, 74% of patients reported at least some problems performing usual activities, 73% of patients were at least moderately anxious or depressed, 51% of patients had at least some problems walking and 41% of patients had at least some problems washing or dressing (Supplementary Fig. 3).

For patients completing the EQ-5D VAS (*n* = 766), the overall mean (SD) EQ-5D VAS score was 54.2 (20.3) (Fig. 4B). Patients currently receiving singlet chemotherapy as 1 L-M and other treatments had the highest and lowest mean EQ-5D VAS scores, respectively. Differences between patients receiving other treatments and all other groups exceeded the MID threshold. By current therapy line (Fig. 4B), the difference observed between EQ-5D VAS scores at 1 L-M and at BSC was the only difference that exceeded the MID.

The overall LCSS-3-IGI (*n* = 762) mean (SD) score was 143.2 (64.5) (Fig. 3B). Patients currently receiving singlet chemotherapy as 1 L-M and other treatments had the highest and lowest mean LCSS-3-IGI scores, respectively (Fig. 3B). Differences observed between singlet chemotherapy as 1 L-M and those receiving singlet chemotherapy as SACT, other treatments and BSC were all above the MID. Considering current therapy line (Fig. 3B), patients currently receiving 1 L-M and BSC had the highest and lowest scores, respectively, and differences above the MID were observed between patients at 1 L-M and those receiving SACT at 2 L+ and BSC.

### Impact on activity

For patients with available LCSS-normal activities data (*n* = 765), the overall mean (SD) score was 51.9 (24.6) (Fig. 3C). Patients receiving BSC and patients receiving singlet chemotherapy as 1 L-M had the highest and lowest scores, respectively. Scores from patients receiving singlet chemotherapy as 1 L-M and those receiving triplet chemotherapy were different to those receiving singlet chemotherapy as SACT, other treatments and BSC, and differences exceeded the MID. Differences exceeding the MID were also observed between patients at 1 L-M and patients receiving SACT at 2 L+ or BSC. For patients completing the WPAI-activity impairment (*n* = 737), the overall mean (SD) percentage of degree of activity impairment was 56.0% (23.2%). With the exception of patients currently receiving triplet chemotherapy (49.3%), all current treatment groups experienced > 50% activity impairment during the past 7 days (Table 2). The percentage of time spent with activity impairment was > 50% across all therapy lines, with patients currently receiving SACT at 2 L+ (59.6%) or BSC (61.2%) experiencing the greatest levels of impairment (Table 2).

**Table 2:** WPAI of MPM patients, stratified by current treatment and current line of therapy at data abstraction, for five EU countries (combined data)

		Current treatment						Current line of therapy			
		Doublet	Triplet	Singlet chemotherapy as 1 L-M	Singlet chemotherapy as SACT	Other treatments	BSC	1-L	1-L M	2 L + SACT	BSC
**Total number of patients with completed PSC who are currently employed**	**107**	**62**	**3**	**14**	**13**	**12**	**3**	**70**	**17**	**17**	**3**
**WPAI Absenteeism**											
n	75	40	2	11	10	9	3	45	14	13	3
Mean (SD), %	36.7 (38.5)	24.9 (33.3)	39.9 (22.1)	57.4 (41.3)	35.2 (40.0)	61.6 (41.8)	45.9 (47.0)	25.0 (32.1)	49.9 (42.0)	60.8 (41.5)	45.9 (47.0)
Missing	32	22	1	3	3	3	0	25	3	4	0
**WPAI Presenteeism**											
n	83	54	3	8	10	6	2	61	11	9	2
Mean (SD), %	47.6 (25.5)	47.0 (27.2)	50.0 (17.3)	50.0 (20.0)	44.0 (29.5)	50.0 (17.9)	60.0 (28.3)	47.2 (26.5)	48.2 (20.4)	46.7 (27.4)	60.0 (28.3)
Missing	24	8	0	6	3	6	1	9	6	8	1
**WPAI Overall Impairment**											
n	58	35	2	6	8	5	2	40	9	7	2
Mean (SD), %	47.4 (24.1)	43.2 (22.6)	64.6 (24.9)	54.1 (14.2)	43.3 (31.8)	60.1 (28.8)	68.5 (20.3)	44.6 (23.4)	52.8 (21.3)	50.7 (32.0)	68.5 (20.3)
Missing	49	27	1	8	5	7	1	30	8	10	1
**Total number with completed PSC**	**767**	**399**	**29**	**89**	**97**	**59**	**94**	**475**	**102**	**96**	**94**
**WPAI Activity Impairment**											
n	737	384	27	84	93	55	94	457	93	93	94
Mean (SD), %	56.0 (23.2)	54.1 (21.4)	49.3 (25.6)	50.8 (21.2)	60.2 (28.0)	64.5 (23.9)	61.2 (23.8)	55.2 (22.8)	51.2 (21.3)	59.6 (25.8)	61.2 (23.8)
Missing	30	15	2	5	4	4	0	0	9	3	0

*Note:* EU countries include France, Germany, Italy, Spain, and the UK

*BSC* Best supportive care; doublet chemotherapy; *MPM* Malignant pleural mesothelioma; others, other therapies; *SACT* Systemic anti-cancer therapy; *SD* Standard deviation; triplet, triplet chemotherapy; *WPAI* Work Productivity and Activity Impairment questionnaire; *1 L* First-line therapy; *1 L-M* First-line maintenance therapy; *2 L + SACT* SACT at second or later lines

## Discussion

This study provides insights into current disease management of MPM patients in routine clinical practice and the humanistic burden of the disease. The patient population represented elderly patients with advanced, unresectable MPM. Patients were mostly men with a history of asbestos exposure. Only one in five patients had a current ECOG PS of 0, indicating that the majority of patients were experiencing some restriction in performance.

The observed treatment patterns generally followed recommended guidelines from ESMO [1] that were available at the time of the study, with platinum+antifolate doublet chemotherapy the most widely used treatment regimen in patients at the time of data abstraction. Previous studies that reviewed treatment patterns covering periods form 2005 to 2013, conducted in France [17], Spain [18] and the UK [19], similarly showed the dominant use of this doublet chemotherapy regimen. Our findings demonstrate that there has been little treatment innovation within the past decade.

A small number of notable differences from ESMO recommendations were observed. Cisplatin is recommended as a platinum+antifolate doublet chemotherapy but a large number of patients were receiving carboplatin. High use of carboplatin due to a lack of tolerability to cisplatin and patients’ older age, has been reported previously [18, 20].

Despite no recommended 1 L-M in the guidelines [1], we found that approximately half of the 534 patients that had completed 1 L went on to receive 1 L-M; 77% of these patients received pemetrexed as a singlet chemotherapy. Outcomes such as progression free survival and treatment response rates were not abstracted in this study. However, progression-free survival benefit and tolerability profile were the most cited reasons for prescribing pemetrexed as 1 L-M. A small number of studies have found that pemetrexed monotherapy was safe and effective (overall survival and time to progression) in MPM [18, 21–23], although progression-free survival benefits were not found in a recent study [24].

A number of combinations for 2 L therapy were noted in our study, with the inconsistency refelecting the fact that there were no approved treatments in the 2 L setting in Europe at this time. We found that nearly half (46.1%) of patients that progressed beyond 1 L received BSC after 1 L. This likely reflects the poor prognosis of MPM patients due to the aggressiveness of MPM tumours [25, 26] and that patients’ treatment goals are the palliation of symptoms in order to maintain quality of life and enable them to live a meaningful and dignified life [3].

An aim of MPM management is for the administration of chemotherapy without delay and before clinical deterioration in those patients who are fit enough to receive it [27]. In this study, a high proportion of patients were diagnosed with advanced disease. Because of the advanced nature of disease, many patients also reported poor HRQoL and high symptom burden, known contributors to poor prognosis [28].

The pattern of results observed in this study suggest that despite receiving treatment for their condition MPM patients experience considerable symptom burden and poor health states. When compared with advanced non-small cell lung cancer (NSCLC) patients, MPM patients have higher symptom burden (LCSS ASBI compared with Iyer et al. [29], − 29.6 vs 42.3), worse health states (EQ-5D UI compared with Wood et al. [30]– 0.51 vs 0.67) and worse HRQoL (LCSS-3-IGI compared with Reck et al., [15] [clinical trial data] – 143.2 vs 193.1). The EQ-5D UI and VAS scores observed in this study were also worse than population norms derived for individuals aged 65–74 years [31]. Each of the differences described above were above the MID threshold for each measure.

We found that HRQoL was poor for 1 L MPM patients. When patients were stratified by line of therapy, HRQoL was poorer in patients at later lines of therapy, suggesting that HRQoL declines further following disease progression. HRQoL declines following disease progression have been reported previously, although previous studies focused on declining emotional well-being and involved far fewer patients [9, 10]. Previous real-world observational studies demonstrated that treating MPM patients with chemotherapy may maintain HRQoL in the short term [32] but clinically meaningful improvements in HRQoL have not been observed. This study found that patients currently receiving 1 L-M experienced meaningfully better health states and HRQoL than patients currently at 1 L and 2 L+. The patients receiving 1 L-M may be expected to be the patients that had experienced a response to treatment from 1 L SACT. This assertion is consistent with the finding that progression free survival benefit was cited as the main reason for choosing 1 L-M. Our findings suggest that HRQoL may be meaningfully improved and at worst maintained for longer if 1 L treatments are able to maintain a response over time. The CheckMate-743 trial data has recently demonstrated that for unresectable MPM patients a 1 L combination of nivolumab plus ipilimumab, had longer overall survival and duration of response, enabled patients to maintain HRQoL for longer and increased time to HRQoL deterioration as compared to platinum+antifolate combination chemotherapy [33]. After a lack of innovation in treatment options in the past decade, the FDA recently approved this combination as a 1 L treatment for unresectable MPM patients [34]. HRQoL should be a key consideration in treatment decisions and additional therapeutic options such as this, with demonstrated maintenance of HRQOL, may be expected to improve patient experience in the absence of a cure.

The several clinically meaningful differences observed across treatment strata (with patients currently receiving singlet chemotherapy as maintenance experiencing better HRQoL) likely reflect the chronicity of treatment rather than treatment efficacy, with the best performing 1 L patients subsequently receiving singlet maintenance treatment. Patients receiving singlet chemotherapy as SACT or other treatments may have been receiving these treatments due to poor performance status and HRQoL at treatment initiation.

Our findings suggest that decrements in HRQoL are caused by declines in multiple domains that impact normal activities, likely caused by high symptom burden. Most MPM patients in this study experienced pain/discomfort, were anxious/depressed and had problems performing their usual activities, effects which are well reported [35]. Walking, washing or dressing also presented a problem for > 40% of patients which may be expected in patients frequently experiencing dyspnea, chest pain and fatigue/weakness. MPM had a high impact on normal daily activities (mean degree of activity impairment = 56%) and impairment was greater than observed in advanced NSCLC patients (LCSS normal acitivies item 51.9 vs 37.5 and 34.5, respectively, at baseline and 71 days after starting combination chemotherapy in Hollen et al., 1999). Consistent with previous findings that healthcare providers underestimate the number of patients with common symptoms and symptom severity of patients with cancer [36], physicians in this study underestimated the proportion of patients experiencing common symptoms.

A key strength of this study was the geographical spread of physicians and their patients, which provided diverse and sizable patient population for evaluating the impact of this rare cancer. These real-world data are representative of MPM patients presenting in routine clinical care and provide treatment patterns and PRO data outside of the clinical trial environment. However, as with all cross-sectional study designs, the current study provides a snapshot of patient status.

A limitation was that the study relied on the accuracy of patients’ medical charts and recall of events by patients attending for physician consultation, although the selection of validated instruments that require short recall time was expected to minimise these effects, with data collected at time of consultation to mitigate against recall bias. Physician and patient inclusion was influenced by their willingness to participate, which had an inherent risk of potential selection bias, however physicians were instructed to abstract data for their next consecutive consulting patients in order to generate a sample reflective of real-world clinical practice. More than 1 in 2 of the patients opportunistically sampled consented to complete a PSC. When comparing the medical record data, we noted several minor but statistically significant differences between the patients that completed a PSC and those that did not. Every effort was made to enable consulting patients to take part in this study and despite these minor differences a heterogenous sample of PSC completers was achieved.

## Conclusions

This study provides evidence that treatments are being prescribed as per available guidance. Despite this, the humanistic burden of MPM is high and MPM patients’ experience important detriment to their HRQoL. Given the poor prognosis for MPM patients and high overall symptom burden of the disease, treatments that are most likely to improve or maintain patients’ HRQoL are those that maintain a response, provide palliation of symptoms and reduce the impact on daily activities. Additional 1 L treatment options are needed as patients experienced poor HRQoL despite receiving treatments administered in line with current guidelines. New treatment options are emerging at 1 L and initial HRQoL findings are positive. To support treatment decision making there is a need for prospective studies comparing the effectiveness of MPM treatments at maintaining/improving HRQoL over time. Improvements in MPM characterisation and diagnostic techniques, such as imaging modalities and biomarkers, are also required to increase the proportion of patients diagnosed with early-stage disease before performance status and HRQoL deteriorates.

## Supplementary Information

**Additional file 1: Supplementary Table 1.** Overview of current treatment groupings used for the current treatment PRO data stratifications.

**Additional file 2: Supplementary Table 2.** Reasons for treatment selection at 1 L and 1 L-M.

**Additional file 3: Supplementary Table 3.** Overview of received treatment regimens, stratified by line of therapy.

**Additional file 4.**

**Additional file 5: Supplementary Fig. 1.** Mean EQ-5D UI (A) and mean EQ-5D VAS scores (B) of MPM patients, stratified by country and by MPM histology. *Note:* The MID for the EQ-5D UI score is 0.08 points, and the MID for the EQ-5D VAS score is 7 points. The population norm is based on individuals aged between 65 and 74 years in each market. Error bars represent 95% confidence intervals. Biph, biphasic; Epi, epithelioid; EQ-5D, European quality of life–5 dimensions; Fra, France; Ger, Germany; UI, utility index; Ita, Italy; MID, minimally important difference; MPM, malignant pleural mesothelioma; Sarc, sarcomatoid; Spa, Spain; UK, United Kingdom; Unk, unknown; VAS, visual analogue scale.

**Additional file 6: Supplementary Fig. 2.** Mean LCSS ASBI (A), mean LCSS-3-IGI (B), and mean Overall Impact on Normal Activities (C) of MPM patients, stratified by country and by MPM histology. *Note:* The MID for the LCSS ASBI is 10 points and the MID for the LCSS-3-IGI is 30 points. Error bars represent 95% confidence intervals. ASBI, average symptom burden index; Biph, biphasic; Epi, epithelioid; Fra, France; Ger, Germany; Ita, Italy; LCSS, Lung Cancer Symptom Scale-Mesothelioma; MID, minimally important difference; MPM, malignant pleural mesothelioma; Sarc, sarcomatoid; Spa, Spain; UK, United Kingdom; Unk, unknown; 3-IGI three-item global index.

**Additional file 7: Supplementary Fig. 3.** EQ-5D UI of MPM patients. *Note:* Patients from EU countries including France, Germany, Italy, Spain, and the UK. Error bars represent 95% confidence intervals. % denotes percentage patients. EQ-5D, European quality of life–5 dimensions; UI, utility index.

## Data Availability

All data relevant to the study are included in the article or uploaded as supplementary information. BMS policy on data sharing may be found at https://www.bms.com/researchers-and-partners/independent-research/data-sharing-request-process.html

## Abbreviations

1 L: First line treatment
3-IGI: 3-item global index
2 L: Second-line treatment
ASBI: Average symptom burden index
BSC: Best supportive care
ECOG PS: Eastern Cooperative Oncology Group performance status
eCRF: Electronic case report form
ESMO: European Society for Medical Oncology
EQ-5D-3L: 3-level EQ-5D questionnaire
FDA: United States Food and Drugs Administration
HRQoL: Health-related quality of life
LCSS-Meso: Lung Cancer Symptom Scale for Mesothelioma
LOT: Line of therapy
MID: Minimally important difference
MPM: Malignant pleural mesothelioma
NCCN®: National Comprehensive Cancer Network® (NCCN®)
NSCLC: Non-small cell lung cancer
PROs: Patient-reported outcomes
PSC: Patient self-completion questionnaire
SACT: Systemic anti-cancer therapy
SD: Standard deviations
UK: United Kingdom
UI: Utility Index
VAS: Visual Analogue Scale
WPAI: Work Productivity and Activity Impairment questionnaire
