# Supplementary material for: Malignant pleural mesothelioma: treatment patterns and humanistic burden of disease in Europe
Source: BMC Cancer. 2022 Jun 23;22:693. doi: 10.1186/s12885-022-09750-7 (PMC9229520; doi:10.1186/s12885-022-09750-7)
Supplement: Supplementary file 1 — Additional file 1: Supplementary Table 1. Overview of current treatment groupings used for the current treatment PRO data stratifications. [file 12885_2022_9750_MOESM1_ESM.docx]

**SUPPLEMENTARY TABLE 1.** Overview of current treatment groupings used for the current treatment PRO data stratifications

| **Treatment group** | **Treatment regimen** | **Treatment** |
| --- | --- | --- |
| GROUP 1  Combination doublet chemotherapy of pemetrexed or raltitrexed with cisplatin or carboplatin | Pemetrexed + platinum | Pemetrexed + cisplatin |
|  |  | Pemetrexed + carboplatin |
|  | Pemetrexed + platinum | Pemetrexed disodium + cisplatin |
|  |  | Pemetrexed disodium + carboplatin |
|  | Raltitrexed + platinum | Raltitrexed + cisplatin |
|  |  | Raltitrexed + carboplatin |
| GROUP 2  Combination triplet chemotherapy (As for Group 1 + an additional agent, e.g., bevacizumab, gemcitabine, immunotherapy) | Pemetrexed + platinum + immunotherapy | Pemetrexed + carboplatin + immunotherapy |
|  |  | Pemetrexed + cisplatin + immunotherapy |
|  | Pemetrexed + platinum + other | Pemetrexed + cisplatin + other |
|  |  | Pemetrexed + carboplatin + other |
|  | Pemetrexed + platinum + other chemotherapy | Pemetrexed + gemcitabine + cisplatin |
|  |  | Pemetrexed + gemcitabine + carboplatin |
|  | Pemetrexed + platinum + bevacizumab | Pemetrexed + cisplatin + bevacizumab |
|  |  | Pemetrexed + carboplatin + bevacizumab |
|  |  | Pemetrexed disodium + carboplatin + bevacizumab |
| GROUP 3  Singlet chemotherapy, groupings here used for singlet chemotherapy as 1L-M, and singlet chemotherapy as SACT | Pemetrexed | Pemetrexed |
|  |  | Pemetrexed disodium |
|  | Other chemotherapy | Gemcitabine |
|  |  | Vinorelbine |
|  | Platinum | Cisplatin |
|  |  | Carboplatin |
|  | Raltitrexed | Raltitrexed |
| GROUP 4  Others, e.g., immunotherapy, gemcitabine + cisplatin + bevacizumab | Immunotherapy | Immunotherapy |
|  | Other chemotherapy + platinum | Gemcitabine + carboplatin |
|  |  | Gemcitabine + cisplatin |
|  |  | Vinorelbine + carboplatin |
|  |  | Vinorelbine + cisplatin |
|  | Bevacizumab + other chemotherapy + platinum | Gemcitabine + cisplatin + bevacizumab |
|  | Pemetrexed + other chemotherapy | Pemetrexed + gemcitabine |
|  | Bevacizumab | Bevacizumab |
|  | Bevacizumab + platinum | Bevacizumab + carboplatin |
|  | Immunotherapy + other chemotherapy | Immunotherapy + gemcitabine |
|  | Two other chemotherapies | Gemcitabine + vinorelbine |
|  | Two platinum therapies | Carboplatin + cisplatin |
|  | Platinum + other | Carboplatin + other |
|  |  | Oxiplatin + raltitrexed |
|  | Raltitrexed + other chemotherapy | Raltitrexed + gemcitabine |
|  | Other | Other |
| GROUP 5  Best supportive care | N/A | Radiotherapy + active drug treatment |
|  |  | Radiotherapy only |
|  |  | Bisphosphonates |
|  |  | Denosumab |
|  |  | Opioids |
|  |  | Analgesics other than opioids |
|  |  | Pleural aspiration/drainage |
|  |  | Non-drug interventions |
|  |  | Watch and wait approach |
|  |  | Other |

*Note:* Patients from EU countries including France, Germany, Italy, Spain, and the UK.

MPM, malignant pleural mesothelioma; N/A, not applicable.
