# Supplementary material for: Malignant pleural mesothelioma: treatment patterns and humanistic burden of disease in Europe
Source: BMC Cancer. 2022 Jun 23;22:693. doi: 10.1186/s12885-022-09750-7 (PMC9229520; doi:10.1186/s12885-022-09750-7)
Supplement: Supplementary file 2 — Additional file 2: Supplementary Table 2. Reasons for treatment selection at 1 L and 1 L-M. [file 12885_2022_9750_MOESM2_ESM.docx]

**SUPPLEMENTARY TABLE 2.** Reasons for treatment selection at 1L and 1L-M

| **Rationale for Treatment Selection** | **Line of Therapy, n (%)** | |
| --- | --- | --- |
|  | 1L (n=1390) | 1L-M (n=273) |
| Has the best evidence and efficacy overall | 611 (44) | 92 (34) |
| It is a treatment I am familiar with | 590 (42) | 98 (36) |
| Progression-free survival benefits | 560 (40) | 111 (41) |
| Manageable side effects profile | 541 (39) | 108 (40) |
| It is the standard of care | 535 (38) | 59 (22) |
| Recommended by national / clinical guidelines | 453 (33) | 66 (24) |
| Overall survival benefit | 407 (29) | 73 (27) |
| Provides rapid onset of action | 382 (27) | 24 (9) |
| Maintains or improves the patients’ quality of life | 284 (20) | 65 (24) |
| Included in hospital formulary | 278 (20) | 26 (10) |
| Achieves partial response | 250 (18) | 27 (10) |
| Low incidence of serious events / severe side effects | 245 (18) | 71 (26) |
| Maintains or improves the patients’ performance status | 228 (16) | 47 (17) |
| Effective in combination | 220 (16) | 11 (4) |
| Strong duration of response | 176 (13) | 71 (26) |
| Convenient dosing regimen | 133 (10) | 25 (9) |
| Safe for long-term use | 115 (8) | 56 (21) |
| Easy to secure reimbursed access | 102 (7) | 13 (5) |
| Convenient method of administration | 99 (7) | 16 (6) |
| Has few contradictions | 90 (6) | 29 (11) |
| Achieves complete response | 89 (6) | 14 (5) |
| Other reason | 11 (1) | - |
| None of the above | 11 (1) | 9 (3) |

*Note:* Patients from EU countries including France, Germany, Italy, Spain, and the UK. More than one reason could be selected per patient.
