# Supplementary material for: Malignant pleural mesothelioma: treatment patterns and humanistic burden of disease in Europe
Source: BMC Cancer. 2022 Jun 23;22:693. doi: 10.1186/s12885-022-09750-7 (PMC9229520; doi:10.1186/s12885-022-09750-7)
Supplement: Supplementary file 3 — Additional file 3: Supplementary Table 3. Overview of received treatment regimens, stratified by line of therapy. [file 12885_2022_9750_MOESM3_ESM.docx]

**SUPPLEMENTARY TABLE 3.** Overview of received treatment regimens, stratified by line of therapy.

| **Treatment Group** | **Treatment Regimen** | **Line of Therapy, n (%)** | | |
| --- | --- | --- | --- | --- |
|  |  | 1L  (n=1390) | 1L-M (n=273) | 2L+  (n=467) |
| Group 1  Combination doublet chemotherapy of pemetrexed or raltitrexed with cisplatin or carboplatin | Pemetrexed + platinum | 1092 (79) | 26 (9) | 13 (3) |
|  | Pemetrexed disodium + platinum | 28 (2) | 1 (0) | - |
|  | Raltitrexed + platinum | 7 (1) | - | - |
| Group 2  Combination triplet chemotherapy (As for Group 1 + an additional agent, e.g., bevacizumab, gemcitabine, immunotherapy) | Pemetrexed + platinum + immunotherapy | 6 (0) | - | - |
|  | Pemetrexed + platinum + other | 2 (0) | - | - |
|  | Pemetrexed + platinum + other chemotherapy | 3 (0) | - | - |
|  | Pemetrexed + platinum + bevacizumab | 44 (3) | 2 (1) | - |
| Group 3  Singlet chemotherapy, groupings here used for singlet chemotherapy as 1L-M, and singlet chemotherapy as SACT | Pemetrexed | 55 (4) | 210 (77) | 16 (3) |
|  | Other chemotherapy | 42 (3) | 13 (5) | 121 (26) |
|  | Platinum | 22 (2) | 3 (1) | 6 (1) |
|  | Raltitrexed | 1 (0) | 1 (0) | 5 (1) |
| Group 4  Others, e.g., immunotherapy, gemcitabine + cisplatin + bevacizumab | Immunotherapy | 9 (1) | 1 (0) | 37 (8) |
|  | Other chemotherapy + platinum | 75 (5) | 4 (1) | 24 (5) |
|  | Bevacizumab + other chemotherapy + platinum | 1 (0) | - | - |
|  | Pemetrexed + other chemotherapy | 1 (0) | 1 (0) | 2 (0) |
|  | Bevacizumab | - | 2 (1) | - |
|  | Bevacizumab + platinum | - | - | 1 (0) |
|  | Immunotherapy + other chemotherapy | - | - | 1 (0) |
|  | Two other chemotherapies | - | - | 6 (1) |
|  | Two platinum therapies | 1 (0) | - | 1 (0) |
|  | Platinum + other | - | - | 2 (0) |
|  | Two platinum therapies + pemetrexed | 1 (0) | - | - |
|  | Raltitrexed + other chemotherapy | - | - | 1 (0) |
|  | Other | - | 9 (3) | 1 (0) |
| Group 5  Best supportive care | N/A | - |  | 230 (49) |

*Note:* Patients from EU countries including France, Germany, Italy, Spain, and the UK.

N/A, not applicable.
