# Supplementary figures and images for: Malignant pleural mesothelioma: treatment patterns and humanistic burden of disease in Europe
Source: BMC Cancer. 2022 Jun 23;22:693. doi: 10.1186/s12885-022-09750-7 (PMC9229520; doi:10.1186/s12885-022-09750-7)

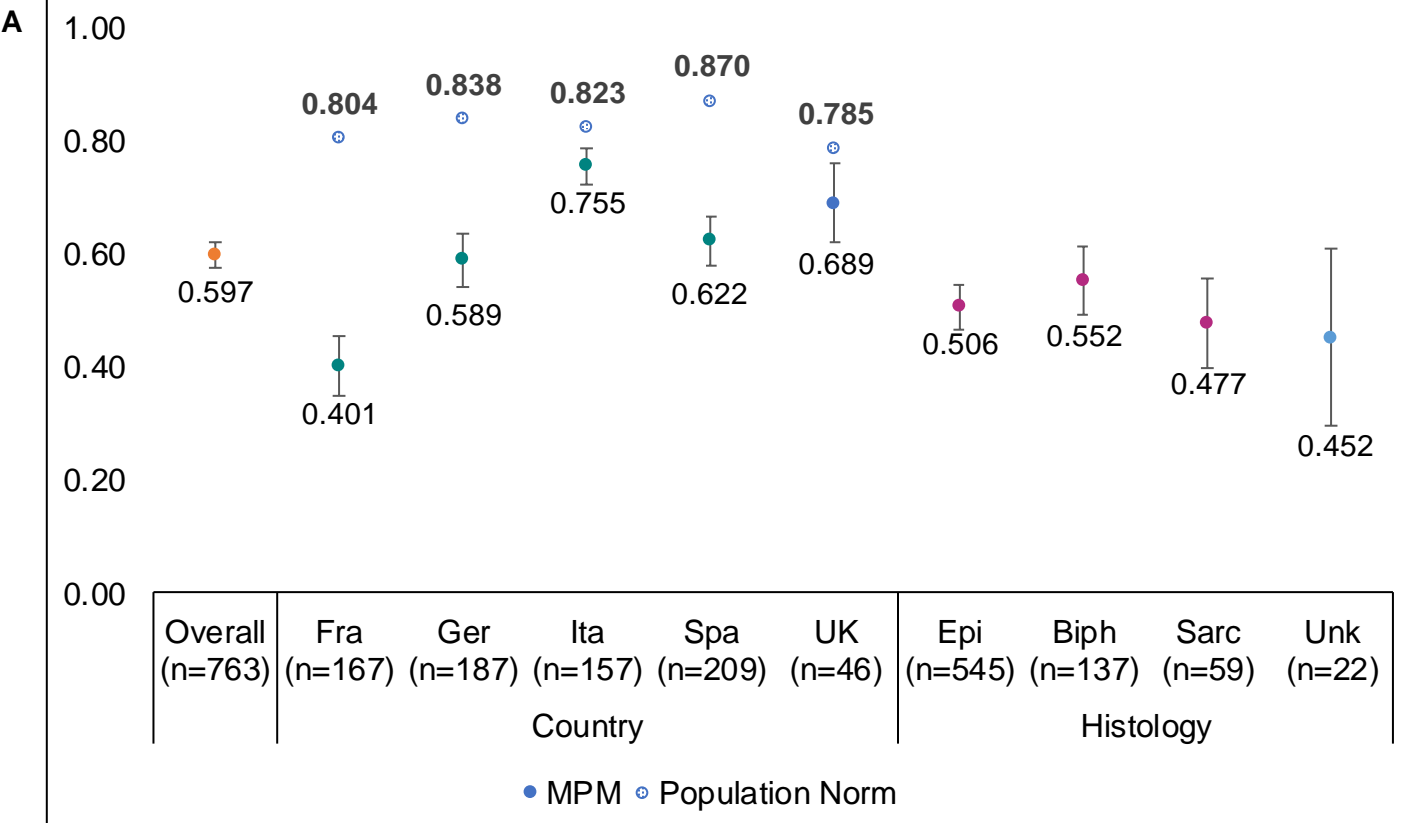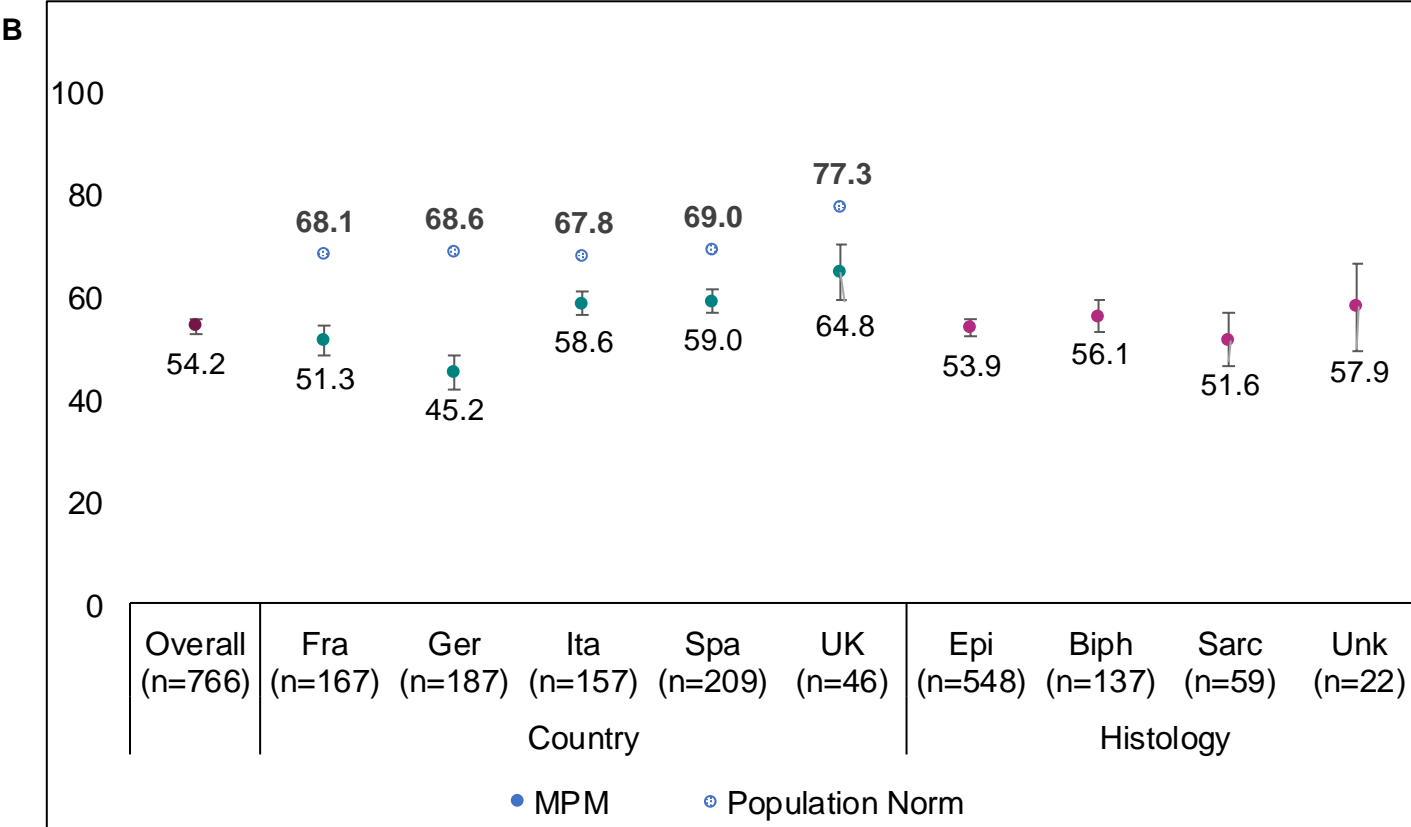

Supplement: Supplementary file 5 — Additional file 5: Supplementary Fig. 1. Mean EQ-5D UI (A) and mean EQ-5D VAS scores (B) of MPM patients, stratified by country and by MPM histology. Note: The MID for the EQ-5D UI score is 0.08 points, and the MID for the EQ-5D VAS score is 7 points. The population norm is based on individuals aged between 65 and 74 years in each market. Error bars represent 95% confidence intervals. Biph, biphasic; Epi, epithelioid; EQ-5D, European quality of life–5 dimensions; Fra, France; Ger, Germany; UI, utility index; Ita, Italy; MID, minimally important difference; MPM, malignant pleural mesothelioma; Sarc, sarcomatoid; Spa, Spain; UK, United Kingdom; Unk, unknown; VAS, visual analogue scale. [file 12885_2022_9750_MOESM5_ESM.pdf]

A

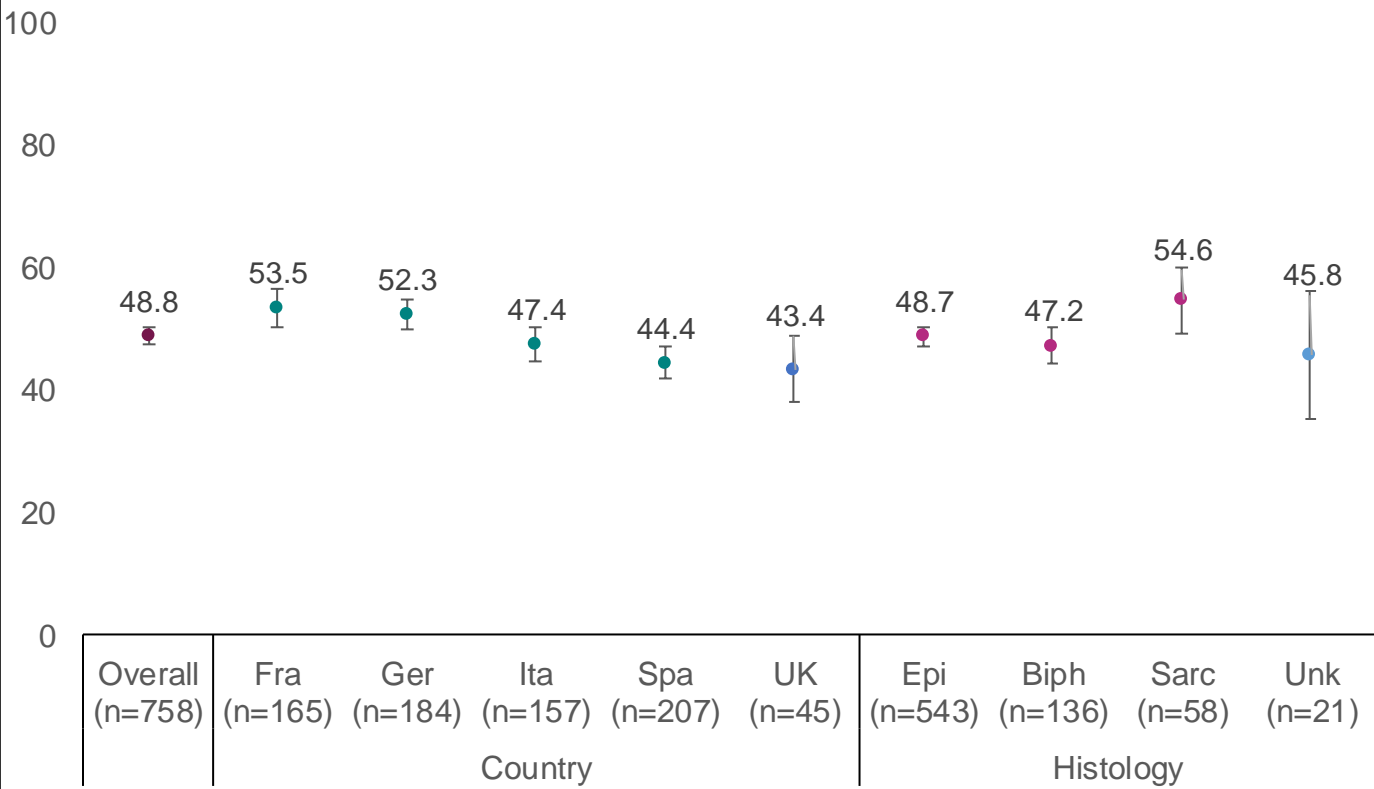

B

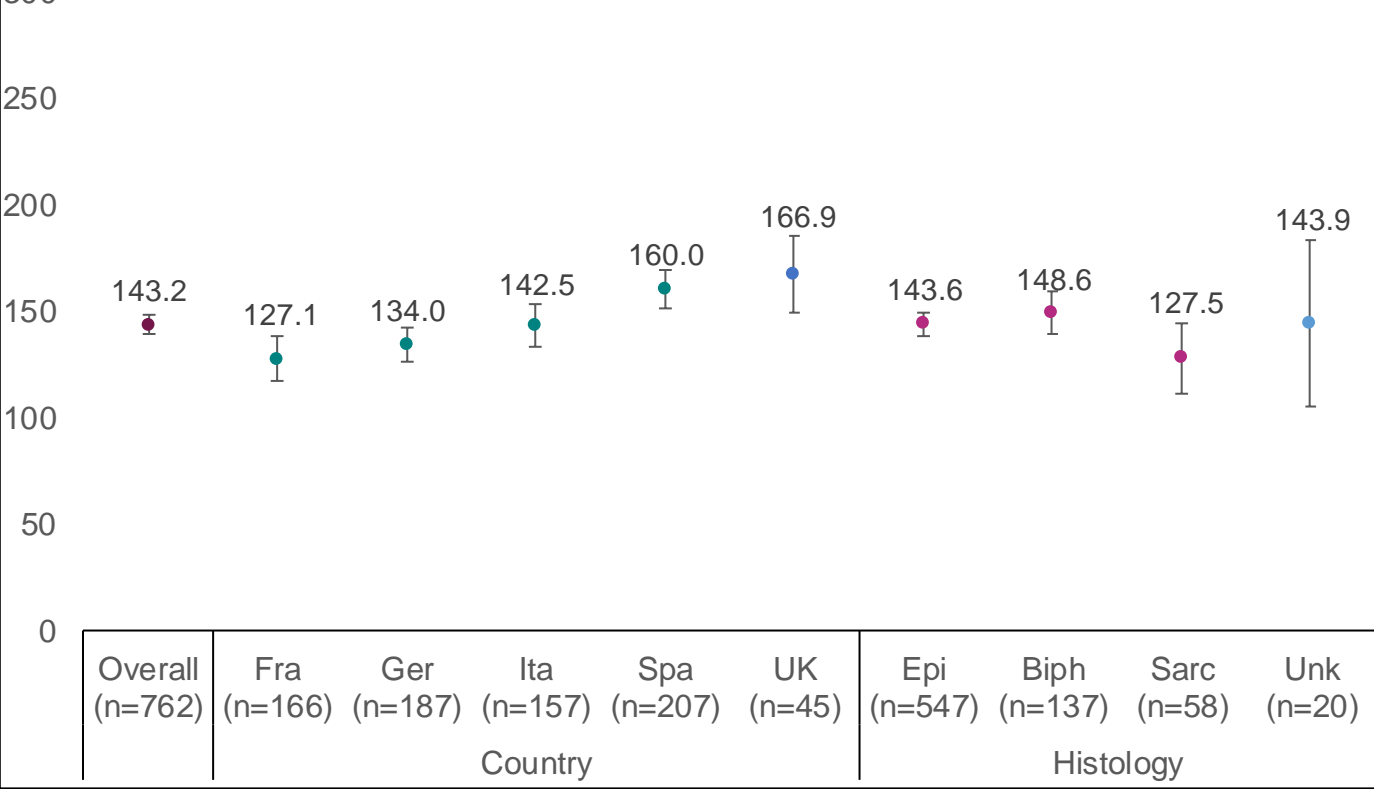

C

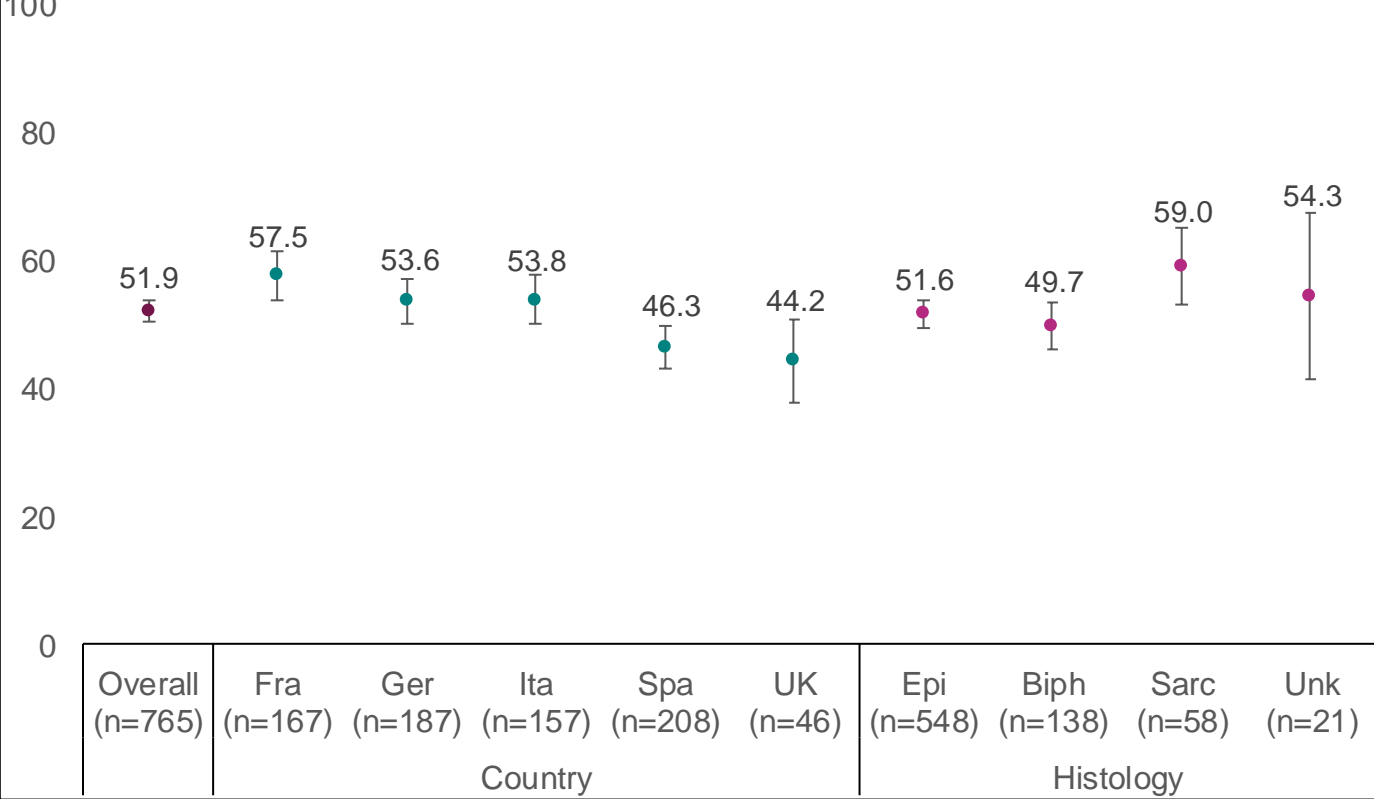

Supplement: Supplementary file 6 — Additional file 6: Supplementary Fig. 2. Mean LCSS ASBI (A), mean LCSS-3-IGI (B), and mean Overall Impact on Normal Activities (C) of MPM patients, stratified by country and by MPM histology. Note: The MID for the LCSS ASBI is 10 points and the MID for the LCSS-3-IGI is 30 points. Error bars represent 95% confidence intervals. ASBI, average symptom burden index; Biph, biphasic; Epi, epithelioid; Fra, France; Ger, Germany; Ita, Italy; LCSS, Lung Cancer Symptom Scale-Mesothelioma; MID, minimally important difference; MPM, malignant pleural mesothelioma; Sarc, sarcomatoid; Spa, Spain; UK, United Kingdom; Unk, unknown; 3-IGI three-item global index. [file 12885_2022_9750_MOESM6_ESM.pdf]
