# Supplementary material for: Malignant pleural mesothelioma: treatment patterns and humanistic burden of disease in Europe
Source: BMC Cancer. 2022 Jun 23;22:693. doi: 10.1186/s12885-022-09750-7 (PMC9229520; doi:10.1186/s12885-022-09750-7)
Supplement: Supplementary file 7 — Additional file 7: Supplementary Fig. 3. EQ-5D UI of MPM patients. Note: Patients from EU countries including France, Germany, Italy, Spain, and the UK. Error bars represent 95% confidence intervals. % denotes percentage patients. EQ-5D, European quality of life–5 dimensions; UI, utility index. [file 12885_2022_9750_MOESM7_ESM.pdf]

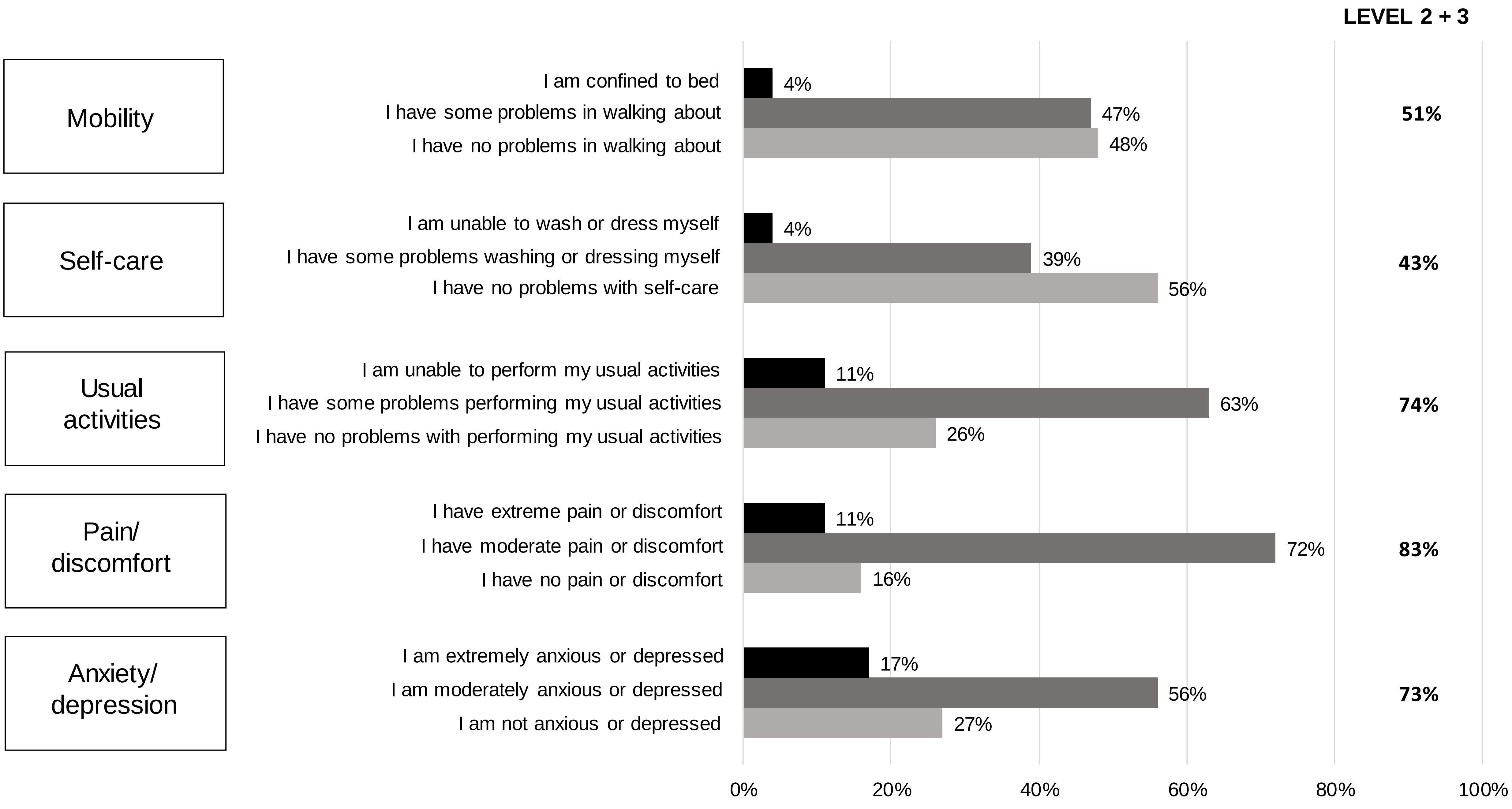

LEVEL 2 + 3
Mobility
I am confined to bed
4%
I have some problems in walking about
47%
I have no problems in walking about
48%
51%
Self-care
I am unable to wash or dress myself
4%
I have some problems washing or dressing myself
39%
I have no problems with self-care
56%
43%
Usual activities
I am unable to perform my usual activities
11%
I have some problems performing my usual activities
63%
I have no problems with performing my usual activities
26%
74%
Pain/ discomfort
I have extreme pain or discomfort
11%
I have moderate pain or discomfort
72%
I have no pain or discomfort
16%
83%
Anxiety/ depression
I am extremely anxious or depressed
17%
I am moderately anxious or depressed
56%
I am not anxious or depressed
27%
73%
0%
20%
40%
60%
80%
100%
